# Yellow nail syndrome with chylothorax after coronary artery bypass grafting

**DOI:** 10.1186/s13019-018-0784-8

**Published:** 2018-09-11

**Authors:** Sarah Waliany, Julia Chandler, David Hovsepian, Jack Boyd, Natalie Lui

**Affiliations:** 1 Stanford University School of Medicine, Stanford, CA USA; 2 Department of Surgery, Stanford University School of Medicine, Stanford, CA USA; 3 Department of Interventional Radiology, Stanford University School of Medicine, Stanford, CA USA; 4 Department of Cardiothoracic Surgery, Stanford University School of Medicine, 300 Pasteur Drive, Stanford, CA 94305 USA; 5 Department of Cardiothoracic Surgery, Division of Thoracic Surgery, Stanford University School of Medicine, Stanford, CA USA

**Keywords:** Yellow nail syndrome, Coronary artery bypass grafting complications, Post-operative chylothorax

## Abstract

**Background:**

Yellow nail syndrome is a rare condition considered secondary to functional anomalies of lymphatic drainage. Yellow nail syndrome is diagnosed through the triad of intrathoracic findings (30% being pleural effusions), nail discoloration, and lymphedema, with any two features sufficient for diagnosis. We report the second case of post-operative yellow nail syndrome.

**Case presentation:**

After coronary artery bypass grafting, our patient presented with chylothorax on post-operative day 13 and yellow toenail discoloration on post-operative day 28, diagnosing yellow nail syndrome. Initial conservative management with pigtail catheter drainage and low-fat diet with medium-chain triglycerides reduced chylous drainage from 350 mL/day on post-operative day 14 to < 100 mL/day on post-operative day 17. However, by post-operative day 18, drainage returned to 350 mL/day that persisted despite attempts to readjust the catheter position, replacement of catheter with chest tube, and transition to total parenteral nutrition and octreotide while nil per os. Lymphangiogram on post-operative day 32 did not identify the thoracic duct or cisterna chyli, precluding embolization. Talc and doxycycline pleurodeses performed on post-operative days 33 and 38, respectively, resolved his chylothorax and nail discoloration.

**Conclusions:**

Both yellow nail syndrome and chylothorax as a complication of coronary artery bypass grafting are rare entities. The proposed mechanism of post-operative chylothorax is iatrogenic injury to thoracic duct or collateral lymphatic vessels. Diagnosing yellow nail syndrome in patients with post-operative chylothorax (through co-existing yellow nail discoloration and/or lymphedema) may suggest predisposition to impaired lymphatic drainage, portending a difficult recovery and potentially indicating need for surgical management.

## Background

Yellow nail syndrome (YNS) is a rare condition of unclear etiology considered secondary to functional anomalies of lymphatic drainage. YNS is characterized by the triad of nail changes, intrathoracic findings (30% being pleural effusions), and lymphedema, with only two of the triad elements required for diagnosis (typically nail discoloration with one of the other findings) [1]. Most cases of YNS have been associated with malignancies, especially lymphoma; autoimmune disorders; immunodeficiencies; endocrine diseases; and others. We report the second case, to our knowledge, of YNS associated with surgery; the first reported case of post-operative YNS was also diagnosed after coronary artery bypass grafting (CABG). We also review the literature on the 41 prior reported cases of chylothorax diagnosed after CABG.

## Case presentation

A 62-year-old man with coronary artery disease underwent four-vessel CABG including left internal thoracic artery (ITA) to left anterior descending artery. The patient was discharged on postoperative day (POD) 6 after an uneventful postoperative course with low chest tube output and trace pleural effusions.

**Fig. 1:**
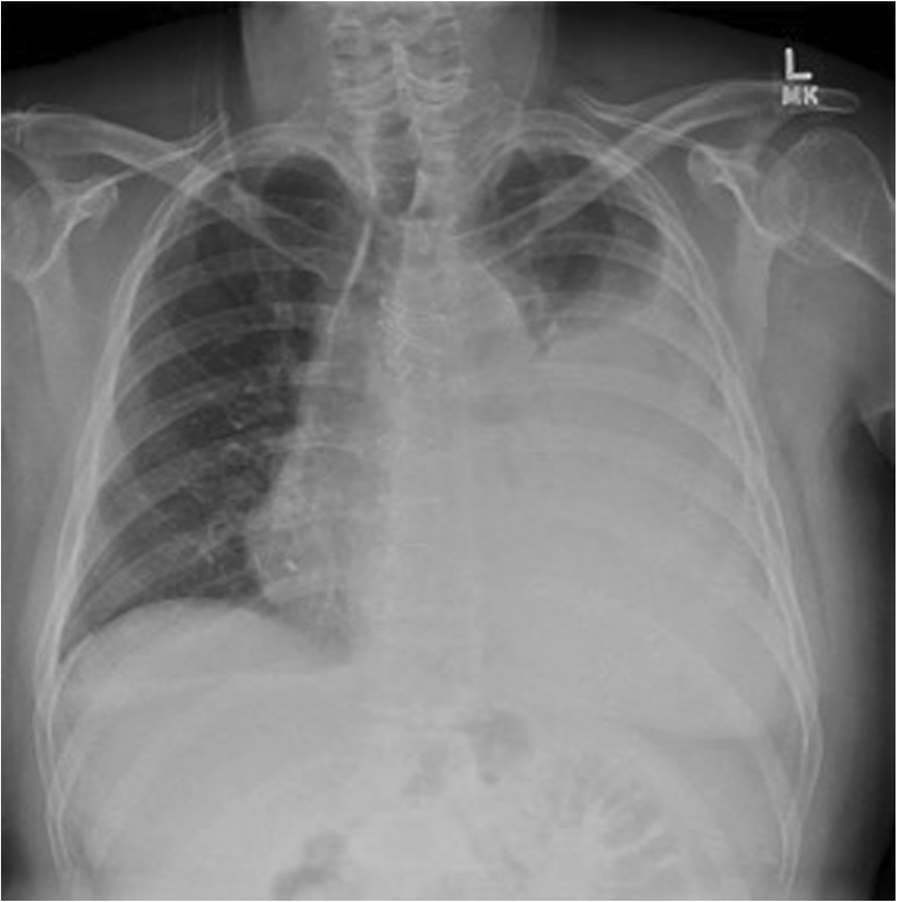
Chest radiograph detected a large pleural effusion over the left lung field on post-operative day 13 after coronary artery bypass grafting

On POD 13, the patient was readmitted after four days of moderate chest pain and exertional dyspnea. Diminished respiratory sounds were noted over the full left lung field. Chest radiograph confirmed a large left pleural effusion (Fig. 1). A left pigtail catheter drained 2.3 l of milky fluid with a triglyceride level of 1604 mg/dL, diagnosing chylothorax.

**Fig. 2:**
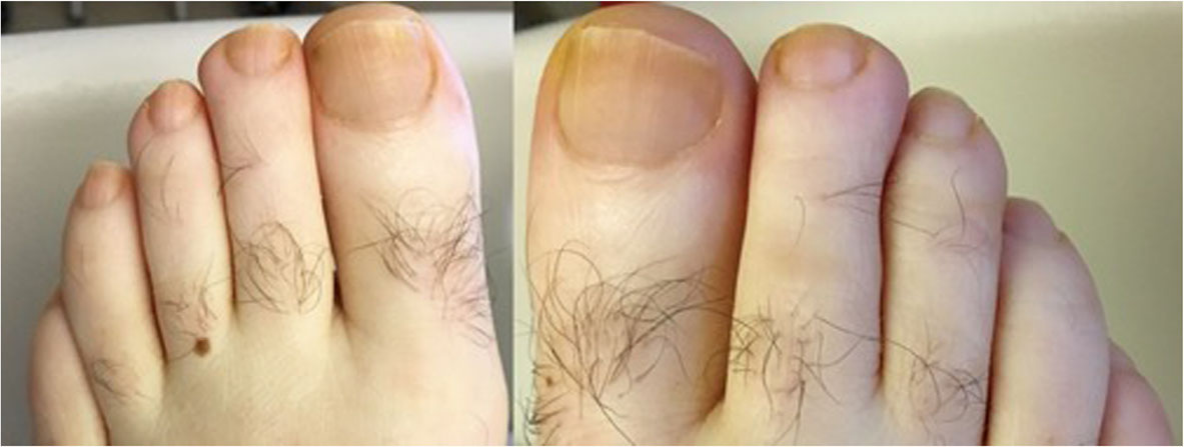
On post-operative day 28 after coronary artery bypass grafting, the patient noted yellow toenail discoloration not present prior to admission

Conservative management was attempted with continued catheter drainage and initiation of a low-fat diet with medium-chain triglycerides on POD 14. Drainage gradually decreased over the next three days to < 100 ml/day, but by POD 18, drainage increased to 350 mL/day that continued for two days despite attempts to readjust the catheter position. On POD 20, the pigtail catheter was replaced with a chest tube, and the patient was transitioned to total parenteral nutrition (TPN) and octreotide while nil per os, but chest tube drainage persisted at 200–360 ml/day. On POD 28, the patient noted new, bilateral yellow toenail discoloration (Fig. 2). The yellow nail discoloration was not associated with any disfiguring features such as nail thickening or separation from nail bed. No lymphedema was found. Presence of yellow nails and chylothorax resulted in diagnosis of YNS.

**Fig. 3:**
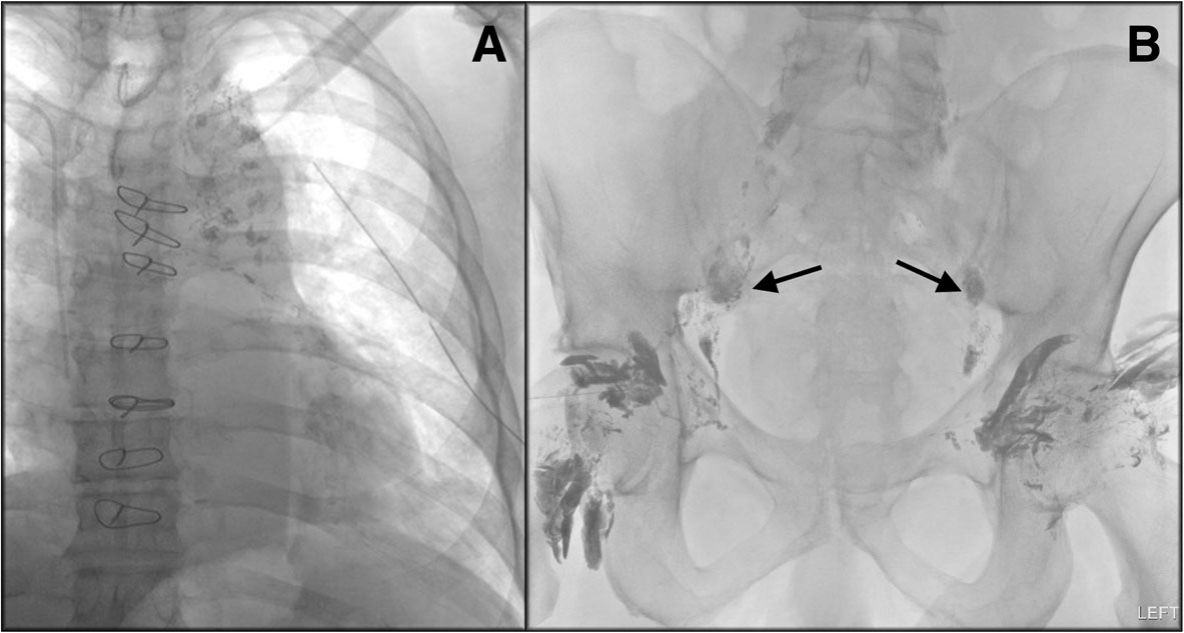
On post-operative day 32, lymphangiogram detected patchy areas of contrast (lipiodol) accumulation in the mediastinum (**a**) without identifying a clearly defined point of lymphatic injury and without identifying the cisterna chyli or thoracic duct (precluding embolization) even though the pelvic lymphatic channels were well-opacified (**b**). Sternal wires and left chest tube were also visible

Lymphangiogram on POD 32 noted well-opacified pelvic lymphatic channels, but cisterna chyli and thoracic duct were not identified (Fig. 3), precluding embolization. Tiny droplets of lipiodol were present in the left pleural space, but the leak location could not be identified.

On POD 33, thoracoscopic left pleurodesis was performed using aerosolized talc (4 g). Prior to talc administration, half-and-half (300 mL) was administered via nasogastric tube, but no chyle leak was found on thoracoscopic examination after two hours. Since lymphangiogram did not identify a thoracic duct, thoracic duct ligation was not attempted. Five days after pleurodesis, since chest tube output remained high (> 200–300 ml/day), doxycycline pleurodesis was performed via existing chest tube. Output subsequently decreased, allowing transition to low-fat diet seven days later and discharge home two days after that.

At 2- and 8-week follow-ups, chest radiograph showed trace pleural effusion with no recurrence of chylothorax. Although still on a low-fat diet at both visits, the patient was gradually increasing fat intake. Yellow toenail discoloration had resolved by the 8-week follow-up.

## Discussion

YNS is diagnosed clinically through the triad of characteristic nail changes, intrathoracic findings (30% being pleural effusions), and lymphedema. Presence of any two features (typically nail discoloration with another finding) is sufficient for diagnosis [1], with the full triad observed in one-third of cases [2]. About 30% of YNS-associated pleural effusions are chylothoraces [3].

Since the first report in 1964 [4], fewer than 400 cases of YNS have been described in the literature. The etiology remains unknown, with prior associations including malignancies (especially lymphoma), immunodeficiency disorders, tuberculosis, diabetes mellitus, thyroid dysfunction, Guillan-Barré syndrome, and others. This is the second report of YNS associated with surgery; the first reported case was also diagnosed after CABG [5].

Although debated, the pathophysiology of YNS is attributed to functional lymphatic defects, with lymphangiogram and lymphoscintigraphy findings of hypoplastic lymphatic vessels [4] and reduced lymphatic drainage in extremities [6]. Nail discoloration results from lipofuscin pigment from oxidation of lipid precursors in soft tissue lymph collections. The conditions associated with YNS are considered to be second insults causing functionally anomalous lymphatics to present clinically through intrathoracic findings, nail changes, and/or edema.

Our report indicates that traumatic disruption of lymphatic vessels may be another trigger causing functionally anomalous lymphatics to manifest clinically as YNS. Intra-operative damage to thoracic duct or other lymphatic tributaries may have overwhelmed the lymphatic network, causing accumulation of chyle in the pleural space as chylothorax and in lower extremity soft tissues manifesting as yellow nails. Although our patient did not have visible lymphedema, a previous study showed that patients with YNS without edema still had significantly reduced lymphatic drainage in lower extremities on lymphoscintigraphy compared with control patients [6]; this suggests that our patient’s lymph collection may have been sufficient to cause lipofuscin accumulation but not edema.

Post-CABG chylothorax is also rare, with 41 other reported cases (Table 1). The proposed mechanism is iatrogenic injury to the thoracic duct or collateral lymphatic vessels. A strong association exists with left ITA harvesting (involving 81.0% of the 42 cases including ours) due to close proximity between the left ITA origin and thoracic duct. Another risk factor is use of electrocautery during harvesting; due to low protein in lymph (compared with blood), electrocautery of lymphatics increases risk of leakage rather than achieving lymphostasis [7].

**Table 1:** Reported cases of chylothorax diagnosed after coronary artery bypass grafting

Reference	Year	Sex	Age	Procedure	L ITA	R ITA	Chylothorax site	POD	Outcome with conservative tx	Octreotide?	Surgical tx
Weber [10]	1981	M	55	CABG	Yes	No	L & M	2	Resolved after 12d	No	None
Zakhour [11]	1981	M	73	CABG	Yes	No	L	90	Resolved after 21d	No	None
Zakhour [11]	1981	M	59	CABG	No	No	L & M	2	Resolved after 5d	No	None
Kshettry [12]	1981	M	51	CABG	No	No	L	30	Resolved after 4d	No	None
Teba [13]	1985	F	51	CABG/MVR	No	No	L	7	Resolved after 17d	No	None
Di Lello [14]	1987	M	53	CABG	Yes	No	L	9	Failed after 31d	No	L thoracotomy-fibrin glue
Czarnecki [15]	1988	F	61	CABG	Yes	Yes	R	42	Failed after 10d	No	R thoracotomy-ligation at diaphragm
Chaiyaroji [16]	1991	F	69	CABG	Yes	No	L	6	Failed after 18d	No	L thoracotomy-ligation at injury
Inderbitzi [17]	1992	M	69	CABG/Redo	UK	UK	L	2	Failed after 21d	No	Left VATS - fibrin glue
Bogers [18]	1993	M	41	CABG	Yes	No	L	1	Failed after 35d	No	L thoracotomy-ligation at injury
Janssen [19]	1994	M	58	CABG	Yes	No	L	35	Failed after 14d	No	L VATS-ligation at injury
Davies [20]	1994	M	48	CABG	Yes	No	L	21	Resolved after 28d	No	None
Wood [21]	1994	M	69	CABG	Yes	No	L	3	Failed after 7d	No	L VATS-ligation at injury
Smith [22]	1994	M	60	CABG	Yes	No	L	14	Resolved after 15d	No	None
Smith [22]	1994	M	47	CABG	Yes	No	L	7	Resolved after 14d	No	None
Zaidenstein [23]	1996	F	70	CABG	Yes	No	L	42	Resolved after 16d	No	None
Felz [24]	1997	F	50	CABG	Yes	No	L	56	Resolved after 59d	No	None
Mohanty [25]	1998	M	56	CABG	Yes	No	L	8	Resolved after 22d	No	Attempted wiring of dehisced sternum on day 9 after diagnosis
Sharpe [26]	1999	F	63	CABG	No	No	L & M	11	Resolved after 14d	No	None
Perez [27]	1999	M	68	CABG/AVR	UK	UK	L	10	Resolved after 10d	No	None
Pego-Fernandez [28]	1999	M	38	CABG	Yes	No	L	90	Resolved after 17d	No	None
Kelly [29]	2000	M	77	CABG	Yes	No	L	18	Resolved after 14d	Yes	None
Fahimi [30]	2001	M	49	CABG	Yes	No	L	UK	Failed after 14d	No	L VATS-fibrin glue
Fahimi [30]	2001	M	51	CABG	Yes	No	L	UK	Resolved after 14d	No	None
Brancaccio [31]	2001	M	64	CABG	Yes	No	L	6	Resolved after 11d	No	None
Abid [32]	2003	M	58	CABG	Yes	No	L	3	Failed after 8d	No	Talc slurry pleurodesis
Riquet [33]	2004	F	59	CABG	Yes	No	L	UK	Failed after 78d	No	L thoracotomy-ligation at injury
Gabbieri [34]	2004	F	67	CABG	Yes	No	L	10	Resolved after 28d	Yes	None
Kilic [35]	2005	F	66	CABG	Yes	No	L	12	Resolved after 10d	Yes	None
Falode [36]	2005	F	68	CABG/ASD	Yes	No	L	3	Failed after 60d	No	L VATS-dry talc pleurodesis
Barbetakis [37]	2005	M	78	CABG	Yes	No	L	27	Resolved after 13d	Yes	None
Halldorsson [38]	2006	F	47	CABG	No	No	R	10	Resolved after 10d	No	None
Choong [39]	2006	M	63	CABG	Yes	No	L	2	Failed after 35d	No	R thoracotomy-ligation at diaphragm
Narayan [40]	2007	F	65	CABG/MVR	Yes	No	UK	1	Failed after 3d	Yes	Median sternotomy-ligation at injury
Paul [41]	2009	M	65	CABG	UK	UK	L	UK	Failed after 14d	UK	R thoracotomy-thoracic duct ligation
Karimi [42]	2010	M	53	CABG	Yes	No	L	2	Resolved after 27d	No	None
Deguchi [43]	2013	F	78	CABG	Yes	Yes	R	3	Failed after 10d	Yes	Median sternotomy-ligation at injury
Altun [44]	2015	M	60	CABG	Yes	No	UK	2	Resolved after 13d	Yes	None
Altun [44]	2015	M	46	CABG	Yes	No	UK	3	Resolved after 14d	Yes	None
Owais [45]	2015	F	76	CABG	Yes	No	L	15	Resolved after 7d	Yes	None
Sabzi [7]	2017	M	43	CABG	Yes	No	L	0	Resolved after 10d	No	None

Abbreviations: *M* = Male, *F* = Female, *ITA* = internal thoracic artery, *L* = Left, *R* = Right, *M* = Mediastinum, *UK* = Unknown, *POD* = “post-operative day” when chylothorax was diagnosed, *d* = days, *tx* = treatment, *CABG* = coronary artery bypass graft, *MVR* = mitral valve replacement, *AVR* = Aortic Valve Replacement, *ASD* = Atrial Septal Defect repair, *VATS* = video-assisted thoracoscopic surgery

An updated adaptation from Halldorsson [38] and Deguchi [43]

Management of post-operative chylothorax starts with conservative measures, including chest tube for effusion drainage. Efforts to decrease chyle production include a trial of low-fat diet with medium-chain triglycerides (absorbed directly into portal system) with transition to TPN and somatostatin or octreotide if output remains high [8]. Conservative management has failed if output exceeds 1 L/day for five days; drainage persists for more than fourteen days; or nutritional status declines. Of the 41 prior cases with post-CABG chylothorax, 26 (63.4%) succeeded with conservative management with all but one resolving in less than 30 days; the other 15 (36.6%) underwent surgery after a median (range) of 14 (3–78) days. In our patient’s case, conservative measures with pigtail catheter drainage and low-fat diet were initially successful at reducing chylous drainage during the first four days of management; however, drainage increased by the fifth day of management likely due to a malfunction of the catheter. Additional conservative measures (chest tube drainage, total parenteral nutrition, and octreotide while nil per os) were continued for an additional 14 days before attempting lymphangiogram; of the 26 reported post-CABG chylothoraces that resolved without surgical intervention, 14 (53.8%) required 14–28 days of conservative management before resolving (see Table 1).

When conservative management fails, thoracoscopic or open thoracic duct ligation with chemical pleurodesis can be done [9]. In chylothorax management at our medical center, it is institutional practice to start with lymphangiogram and minimally-invasive thoracic duct embolization before attempting surgical thoracic duct ligation as our Department of Interventional Radiology has a history of success with the less invasive procedure; in the event that duct embolization fails, lymphangiogram may guide future surgical measures. In our patient’s case, the lymphangiogram guided the surgical team to attempt pleurodesis without thoracic duct ligation since the thoracic duct was not identified.

Although this is the second report, to our knowledge, of post-CABG yellow nail syndrome, it is possible that the other YNS features (yellow nails and lymphedema) have previously been missed in patients with post-CABG chylothorax since the findings are subtle. Other cases may have been misdiagnosed as fungal infections. In our patient’s case, the close temporal association between the development of chylothorax and appearance of yellow nail discoloration and the temporal proximity of their resolution were more consistent with YNS. Furthermore, the appearance of his nails was less consistent with onychomycosis. Other than yellow discoloration, his nails had no disfiguring features such as thickening, chipping, or separation from the nail bed; his nails also demonstrated a uniform yellow discoloration without black debris. Overall, the appearance of his nails was not consistent with any of the major subtypes of onychomycosis (such as distal and lateral subungual; proximal subungual; endonyx subungual; superficial; or total dystrophic onychomycosis) [10].

Although diagnosing YNS does not alter management, we propose that concurrence of chylothorax with yellow nails and/or lymphedema may suggest predisposition to impaired lymphatic drainage and serve as a marker of degree of lymphatic leakage, portending a difficult recovery course and potentially indicating need for surgical intervention. As our patient’s recovery was challenging (failing conservative treatment and requiring both talc and doxycycline pleurodeses), it is possible that he had a lymphatic aberrancy predisposing to YNS, with intraoperative thoracic duct injury serving as the second insult that caused YNS to manifest clinically.

## Conclusions

Chylothorax as a complication of coronary artery bypass grafting is rare, with the most likely mechanism being iatrogenic injury to the thoracic duct or collateral lymphatic vessels. During management of post-operative chylothorax, diagnosis of yellow nail syndrome (through concurrent presence of yellow nail discoloration and/or lymphedema) may suggest a predisposition to impaired lymphatic drainage, potentially indicating the need for surgical management.

## Abbreviations

CABG: Coronary artery bypass grafting
ITA: Internal thoracic artery
POD: Post-operative day
TPN: Total parenteral nutrition
YNS: Yellow nail syndrome
